# Ceramic MEMS Designed for Wireless Pressure Monitoring in the Industrial Environment

**DOI:** 10.3390/s120100320

**Published:** 2011-12-29

**Authors:** Marko Pavlin, Darko Belavic, Franc Novak

**Affiliations:** 1 In.Medica d.o.o., Levicnikova 34, 8310 Sentjernej, Slovenia; 2 Jozef Stefan Institute, Jamova 39, 1000 Ljubljana, Slovenia; E-Mails: darko.belavic@ijs.si (D.B.); franc.novak@ijs.si (F.N.)

**Keywords:** energy harvesting, pressure measurement, thick film sensors

## Abstract

This paper presents the design of a wireless pressure-monitoring system for harsh-environment applications. Two types of ceramic pressure sensors made with a low-temperature cofired ceramic (LTCC) were considered. The first type is a piezoresistive strain gauge pressure sensor. The second type is a capacitive pressure sensor, which is based on changes of the capacitance values between two electrodes: one electrode is fixed and the other is movable under an applied pressure. The design was primarily focused on low power consumption. Reliable operation in the presence of disturbances, like electromagnetic interference, parasitic capacitances, *etc.*, proved to be contradictory constraints. A piezoresistive ceramic pressure sensor with a high bridge impedance was chosen for use in a wireless pressure-monitoring system and an acceptable solution using energy-harvesting techniques has been achieved. The described solution allows for the integration of a sensor element with an energy harvester that has a printed thick-film battery and complete electronics in a single substrate packaged inside a compact housing.

## 1. Introduction

Micro-electro-mechanical systems (MEMS) can be fabricated with a variety of technologies and from a wide range of materials. MEMS are normally made by micro-machining silicon, but in some applications ceramic materials are a very useful alternative, especially in harsh environments and at high temperatures. The laminated 3D structures made using low-temperature cofired ceramic (LTCC) are especially practical for so-called Ceramic MEMS [1–7].

One of the biggest segments of the MEMS market is pressure sensors. The market is currently dominated by silicon pressure sensors, but in some demanding applications thick-film technology and ceramic materials can be used for the fabrication of sensor systems, *i.e.*, ceramic or thick-film pressure sensors. In comparison with semiconductor sensors they are larger, more robust and have a lower sensitivity, but they have a high resistance to harsh environments. Low-temperature cofired ceramic (LTCC) technology and materials are suitable for forming a three-dimensional (3D) construction of pressure sensors and thick-film technology and the materials are used for the creation of electronic interconnections, capacitor electrodes, piezoresistors and other resistors [4–8].

In typical wireless pressure-sensor systems, the power sources are autonomous and mostly based on different types of energy harvesting or on batteries. In both cases, the level of power consumption has a significant influence on the dimensions and the complexity of the system, and on the required frequent charging in the case of the battery power supply. Therefore, the power consumption of the autonomous operating sensors should be minimized as much as possible, not only in the electronic circuitry (signal conditioning, microcontroller, wireless transceiver, *etc.*) but also in the sensing element or transducer. Another very important reason for reducing the power consumption of the sensing element relates to the high power dissipation in the sensing element that generates heat. More heat increases the warm-up time of the sensor and decreases the stability of the sensor’s characteristics [9].

Wireless pressure monitoring [10–12] in the industrial environment must satisfy many different requirements [13–15]. This paper is focused primarily on two aspects. The first is the design and fabrication of the sensing element (transducer) for harsh-environment applications. The second is low power consumption and an effective energy management of the system. The energy management includes how to obtain energy from the environment (energy harvesting), energy storage and/or an autonomous energy source and the low energy consumption of all the components of the system, including the sensing element. The paper is organized as follows. In the next section, basic characteristics of the two sensor candidates for harsh-environment applications are briefly outlined. Their design is described in more details in [8]. Integration into an autonomous wireless-sensor node is described in Section 3, followed by the implementation and experimental results report in Sections 4 and 5. In Section 6 the conclusions are drawn.

## 2. Sensor Device

### 2.1. Construction of LTCC Pressure Sensors

**Figure 1.**
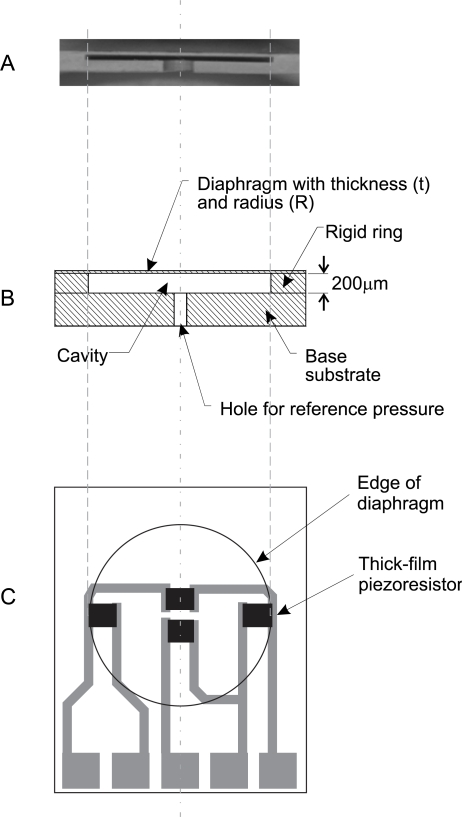
Photograph **(a)**, cross-section **(b)** and top-view **(c)** of the construction of a LTCC pressure sensor.

LTCC technology and materials are suitable for making the ceramic structure of a thick-film pressure sensor, which can work in a wide temperature range and in different media (gasses, liquids) [2,8,16,17]. This structure consists of a circular, edge-clamped, deformable diaphragm that is bonded to a rigid ring and the base substrate. In the base substrate is the hole for the applied reference or differential pressure. These elements form the cavity of the pressure sensor. The depth of the cavity is especially important for the capacitive type of pressure sensor and depends on the thickness of the rigid ring. The cross-section and the top-view of the LTCC construction of the ceramic pressure sensor are schematically shown in Figure 1.

**Figure 2.**
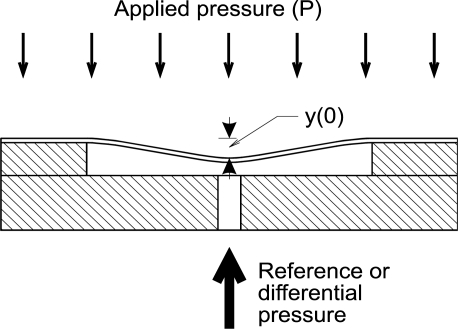
Cross-section of the construction of the LTCC pressure sensor under an applied pressure *P*, which causes the deflection *y*(0) in the middle of the diaphragm.

The structure in Figure 1 was constructed for measuring pressures in the range from 0 to 100 kPa, and for a burst pressure of about 400 kPa. For these characteristics we designed the diaphragm with a thickness of 200 μm and with a radius of 4.5 mm. The depth of the cavity is about 80 μm when the structure is designed for a capacitive type of pressure sensor. It should be as low as possible, but the technology limitations are around 50 μm. For high volume stable production, the 80 μm was chosen as optimal tradeoff between manufacturability and required performance. The LTCC foil used for the 80 μm cavity was DuPont 951PP, which is 100 μm foil when unfired. After firing the thickness shrinks to 80 μm. When the structure is designed for a piezoresistance type of pressure sensor, the functional window is deeper, because the diaphragm should be suspended only at the edges and gap size is not important, therefore it is equal to standard LTCC raw material thickness 200 μm. The LTCC foil used for 200 μm membranes was DuPont 951PX. The cross-section of the cavity within the 3D LTCC structure fabricated with LTCC materials is shown in the above figure. The applied pressure on the ceramic pressure sensors deforms the flexible and deformable diaphragm. The deformation is presented in Figure 2.

The construction, the dimensions and the material properties of the sensor structure influence the sensors’ characteristics for all types of ceramic pressure sensors. The dependence of the geometry and the material properties of the LTCC construction on the deflection of an edge-clamped deformable diaphragm under an applied pressure are described by Equation (1):

(1) $$y\left(r\right)\;=\frac{3P\;\left(1-\nu^{2}\right)\;{\left(R^{2}-r^{2}\right)}^{2}}{16\;E\;t^{3}}$$

where the deflection *y* at the position *r* from the centre of the diaphragm is a function of the applied pressure *P*, the material characteristics of the diaphragm (Young modulus *E*, and Poisson’s ratio *ν*), and the dimensions of the diaphragm (thickness *t*, and radius *R*).

### 2.2. Piezoresistive Ceramic Pressure Sensor

**Figure 3.**
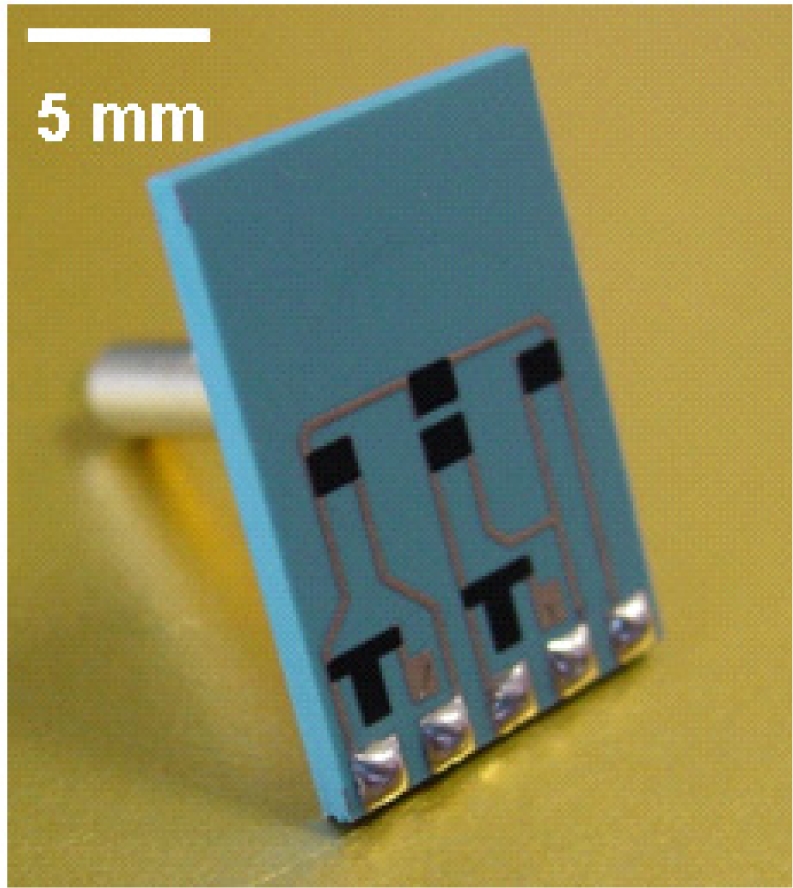
The piezoresistive ceramic pressure sensor.

**Figure 4.**
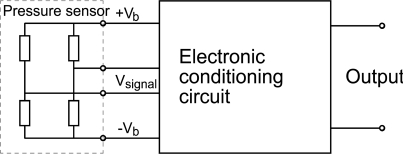
The piezoresistive sensing element is connected to the electronic conditioning circuit.

A piezoresistive ceramic pressure sensor (Figure 3) is based on the piezoresistive properties of the thick-film resistors that are screen-printed and fired onto the deformable diaphragm. The piezoresistive ceramic pressure sensor has four thick-film resistors, which act as strain gauges and transduce a strain into an electrical signal. The sensing resistors are located on the diaphragm so that two are under tensile strain, and two are under compressive strain. These four resistors are electrically connected in a Wheatstone-bridge configuration and excited with a stabilised bridge voltage. The Wheatstone-bridge is integrated with the electronic conditioning circuit in one single ceramic substrate (Figure 4).

The resistances of the sensing resistors connected in a Wheatstone-bridge and the bridge voltage (V_b_) have a direct influence on the power consumption. A lower bridge voltage causes a lower current and therefore a lower power consumption, but the value of the output signal is also lower. The power consumption can also be reduced by the use of a resistor with a high resistance (high sheet resistivity). In this case the sensitivity is slightly higher but the signal-to-noise ratio is lower. The lower bridge voltage and/or the high resistance cause another problem—the sensor becomes more susceptible to the parasitic effects and other interferences.

The values of the resistivity of piezoresistors can be between a few hundred ohms and up to a few hundred thousand ohms. The values of the bridge voltage can be between one volt and a few tens of volts. Therefore, the values of the power consumption can be in a very wide range. However, a typical value of the resistances of the piezoresistors is 10 kΩ and a typical value of the bridge voltage is 5 V. These parameters result in a power consumption of about 2.5 mW.

### 2.3. Capacitive Ceramic Pressure Sensor

**Figure 5.**
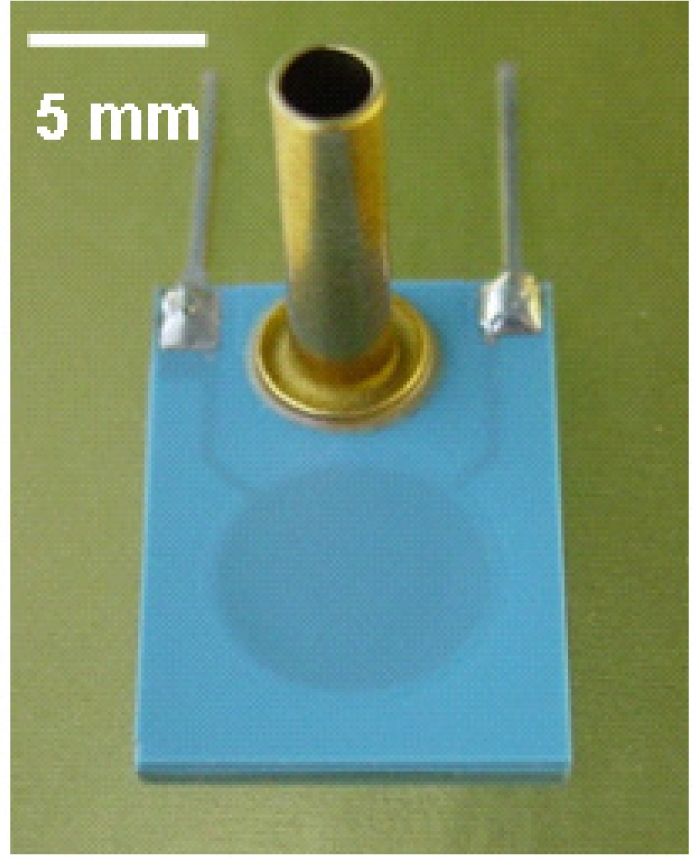
The capacitive ceramic pressure sensor.

The capacitive ceramic pressure sensor (Figure 5) is based on the fractional change in capacitance induced by the applied pressure. The capacitance change is due to the varying distance between the electrodes of the air-gap capacitor. These electrodes are within the cavity of the LTCC structure (Figure 1). The bottom electrode of the capacitor is on the rigid substrate and the upper electrode is on the deformable diaphragm. The areas of the electrodes and the distance between them define the value of the initial capacitance (C_0_) of the capacitive pressure sensor. The distance between the electrodes is calculated from the cavity depth and the thickness of both electrodes.

**Figure 6.**
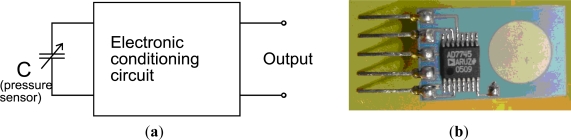
The capacitive sensing element integrated with the electronic conditioning circuit: (**a**) block diagram; (**b**) prototype with surface mounted signal conditioning circuit.

First, the general outline dimensions are defined. Within the given outline, the cavity supporting structure is constructed. The geometry depends on the required pressure range and assembling conditions. Generally, a sensor for high pressures requires more rigid supporting structure, and plastic housing also requires more rigid sensor element compared to a sensor packaged in an alluminium housing. When the membrane size is determined, the gap is calculated first from the capacitor calculation, and then a simulation is run using the estimated geometry. The design is optimized using simulations. Finally, a batch of different designs is produced and evaluated. For reference, a capacitive ceramic pressure sensor (as shown in Figure 6) with 80 μm air gap, 100 μm membrane and 1 cm^2^ electrodes/membrane area has nominal capacitance between 5 pF to 15 pF with capacitance change of 100 fF over 1,000 mBar full scale input pressure.

The capacitive ceramic pressure sensor is integrated with the electronic conditioning circuit with a frequency output in most cases (Figure 6). In this case the typical output frequency is between 10 and 100 kHz, and this varies according to the applied pressure. The power consumption of the sensing element for the capacitive ceramic pressure sensor is very low and depends mostly on the values of the operating frequency and the voltage, as well as the capacitance of the sensing element. Our test samples have a capacitance of around 8 pF, an operating voltage of 1 V, and an operating frequency of 10 kHz. These parameters result in a power consumption of about 0.5 μW. The power consumption was calculated from the impedance and the applied voltage. The values of working frequency and voltage are determined by the electronic signal-conditioning circuit. If we reduce the values of any mentioned parameters the power consumption of the sensing element is lower. But at the same time the pressure sensitivity is lower and the problems with the quality of the output signal and also with the parasitic capacitance are more serious.

### 2.4. Benchmarking of the Sensing Element

The capacitive ceramic pressure sensor has lower power consumption (0.5 μW) in comparison with the piezoresistive sensor (2.5 mW). It is very sensitive to any disturbances, like parasitic capacitances, electromagnetic interference, temperature, humidity, *etc*., [18]. Because of this effect the capacitive pressure sensor is less suitable for use in most prevalent applications for demanding industrial environments. On the other hand, the power consumption of piezoresistive ceramic pressure sensors can be easily controlled by the values of the Wheatstone-bridge resistances. Therefore, the piezoresistive ceramic pressure sensors with a high bridge impedance were chosen for use in a wireless pressure-monitoring system in an industrial environment. Typical characteristics are shown in Table 1.

## 3. Integration into Autonomous Wireless-Sensor Node

**Figure 7.**
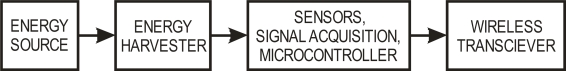
The block diagram of autonomous wireless node.

A typical energy-harvesting wireless-sensor node configuration usually consists of a (free) energy source, an energy harvester, sensors with ADC converters, amplifiers, a microcontroller and a low-power radio communication, as shown in Figure 7.

Energy sources such as a thermoelectric generator (TEG), a small indoor solar cell, a piezoelectric generator, a thermopile or similar supply electrical energy to the energy harvester. These small, sparse and low-power energy bursts with limited peaks are then converted into a usable form to power downstream circuits, such as the low-power sensing elements, the analogue-to-digital converter and a low-power microcontroller. The microcontroller sleeps with an ultra-low-power consumption, usually below 1 μA and periodically wakes up to supply a sensor, takes a reading from it and prepares this data for transmission via a low-power wireless transceiver [19].

**Figure 8.**
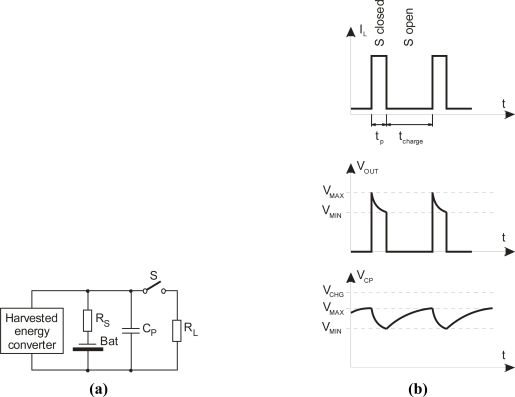
(**a**) Schematic layout of power supply for pulsed operation of a wireless sensor, (**b**) typical internal voltages during pulsed operation (I_L_ = Load current-pulse, V_OUT_ = supply output voltage, V_CP_ = Voltage across storage capacitor C_P_).

Each circuit system block in this chain has its own unique set of constraints. It is essential to design a high-efficiency harvester and power management to keep the amount of time required to power up the complete system to a minimum. Longer power-up times increase the time intervals between taking a sensor reading and transmitting this data. Proper design of a pressure sensor is of great importance here. Many conventional designs are not suitable for autonomous wireless sensors. Quiescent currents in individual blocks shown in Figure 7 define the lower usable limit of an energy-harvesting source. It must first supply the current needed for the system’s self-operation in sleeping mode before it can use any excess to supply and store this power to supply the outputs. A microcontroller with a periphery and RF link normally draws short pulse currents in the couple-of-tens-of-mA range when active. The energy-harvesting process collects available energy from the surrounding environment and stores it in an internal storage device [Figure 8(a)]. Some sources reported in [20] are listed in Table 2.

The operating principle of the energy-harvesting power management is shown in [Figure 8(b)]. The power is collected during sleep-mode operation and stored in a rechargeable battery. The batteries used in such systems are small; sometimes they are a thin film with large internal resistance R_S_, which limits the peak currents in active mode. The simplest solution to overcome this problem is to place a low-Equivalent Series Resistance (low-ESR) capacitor across the main energy-storage device, which is charged during normal charge cycles and can deliver high current pulses. The following key parameters should be defined to calculate the required capacitance and the minimal latency time:

- Storage device internal resistance R_S_,
- Average load resistance R_L_,
- Maximum voltage to which the capacitor must be charged prior to delivering the next current pulse V_MIN_,
- Average current-pulse amplitude and duration I_L_, t_P_,
- Minimum supply voltage at the system input V_MAX_.

## 4. Implementation

The performance of the sensor system from Figure 7 depends on the selection of components for each building block. For our testing system setup we had a choice among different microcontrollers and radio solutions (Table 3). Notice that the Zarlink radio module has the lowest Rx and Tx current. The final selection was a MSP430 microcontroller and a Zarlink radio module due to the flexibility offered by two chip solution which proved to be most suitable for our application.

**Figure 9.**
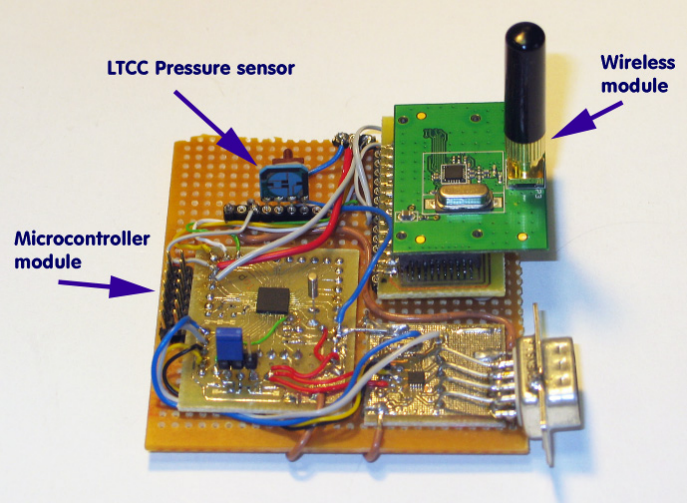
Low-power wireless pressure-monitoring system with a piezoresistive LTCC-based pressure sensor.

**Figure 10.**
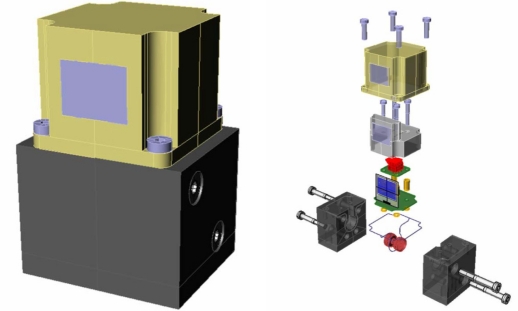
Industrial grade packaging for the target sensor.

The wireless pressure-monitoring system built on a piezoresistive LTCC-based pressure sensor is shown in Figure 9. The data acquisition and processing were done with an MSP430F2350 microcontroller. The wireless interface was based on a CC2500 transceiver with TI’s simplicity low-power radio frequency (RF) communication stack. For real industrial application, proper housing is required with circuit layout redesign to accommodate the dimensions of a particular model, shown in Figure 10.

## 5. Experimental Results

**Figure 11.**
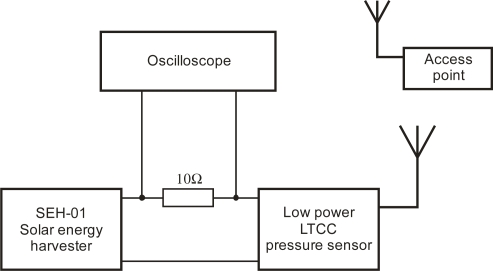
Low-power testing system for LTCC-based pressure sensor.

Test results were obtained by testing the system shown in Figure 11. We used the solar-energy harvester module SEH-01 from the TI development system. It uses photovoltaic energy from an indoor solar cell that converts ambient light into electrical energy. The low voltage from the solar cell is handled by an EnerChip thin-film battery solution. The module can provide a 100-μAh capacity with an average output power of 350 μW for a 1,000-lux light source.

The power consumption of the pressure-sensor module with an LTCC-based sensing element was calculated by measuring the voltage drop with an oscilloscope over the shunt resistor between the energy harvester and the sensor electronics.

**Figure 12.**
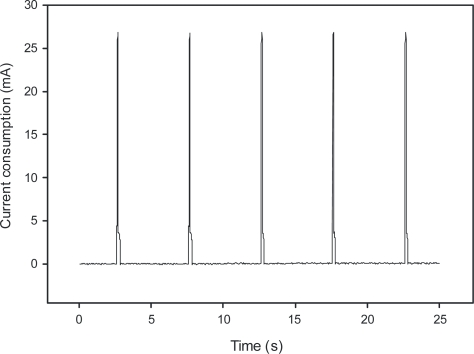
Power consumption of a low-power LTCC sensor over 25 s time.

The LTCC-based pressure sensor sends data to the access point every 5 s. The access point for the testing purposes was USB dongle connected to a PC, used as a pressure monitoring instrument. A custom application was prepared to display and store received readouts. The current bursts are shown on the oscillogram in Figure 12.

The diagram in Figure 12 shows the magnitude and duty cycle of the sensor’s power consumption. The average sleep-mode power consumption depends on the current draw between bursts. Increasing the time between the active periods could result in longer survival periods without any available power, e.g., when the ambient light conditions are dark.

**Figure 13.**
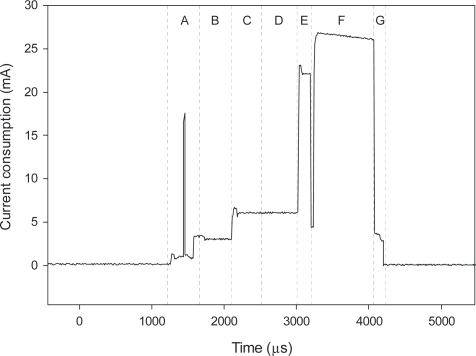
Single current spike anatomy.

The inspection of the anatomy of a single current spike is possible by increasing the sampling frequency. Individual events during the active mode are shown in Figure 13. This is a good base for analyzing the contributions of different hardware parts and software events to the overall power consumption in active mode. Unfortunately, we had no influence on some parts of the radio communication, since the RF software stack was provided as a library and therefore abstracted from the final implementation.

Operation events and their power consumption are listed in Table 4. Total energy consumption in active mode is about 40 μAs, while in the sleep mode the circuit consumes 1.8 μA. The achieved solution provides an energy efficient platform for coin-battery operated products.

## 6. Conclusions and Future Work

The power consumption of pressure-sensing elements (transducers) depends primarily on the sensing principles, as well as on the technology and the materials used. LTCC technology offers a good opportunity to significantly minimize the power consumption of industrial wireless pressure sensors. Our results show that when a low-power LTCC-based sensing element is used, it represents only 0.1% of the total power consumed when in active mode. If a conventional silicon pressure sensor was used, this share would increase to 48.6%, partially due to a much higher current being drawn from the sensor and secondly, as an consequence of the longer warm-up and setup times due to the lower performance power-up behavior. Longer active times then increase the overall power consumption, because in the active mode not only is the sensor powered, but also other components, like the microcontroller and, partially, the RF module.

Our pressure-monitoring system was developed for industrial application with an operating temperature range from −25 °C to +125 °C. The prototype was implemented with an energy harvester from the evaluation module. It shows the practical use of solar-energy harvesting and thin-film battery use. Both are compatible with LTCC technology. In the future, this will open up new possibilities for the integration of a sensor element with an energy harvester that has a printed thick-film battery and complete electronics in a single substrate packaged inside a very compact housing.

The presented ceramic MEMS is the first building block of a platform for autonomous industrial sensors and has all the essential characteristics required for integration into harsh industrial environments. The compact, single-substrate design leaves plenty of room for additional electromagnetic protection circuitry and robust housing, which at the end forms a miniature industrial pressure transducer. Future work will be focused on encapsulation of the presented solution into ingress-protected, electromagnetically compatible housing for specific target applications.

## Figures and Tables

**Table 1.** Benchmarking of the sensing element.

**Parameter**	**Capacitive**	**Conventional Piezoresistive**	**High resistance Piezoresistive**
Power consumption	0.5 μW	2.5 mW	<1 μW
Noise	High	Low	High
Technology	Difficult	Easy	Easy
Environment impact on sensor	High	Low	Low
Signal conditioning demands	High	Low	Low

**Table 2.** Energy-harvesting estimates (as reported in [20]).

**Energy Source**	**Harvested Power**
Vibration/Motion	

Human	4 μW/cm^2^
Industry	100 μW/cm^2^

Temperature Difference	

Human	25 μW/cm^2^
Industry	1–10 mW/cm^2^

Light	

Indoor	10 μW/cm^2^
Outdoor	10 mW/cm^2^

RF	

GSM	0.1 μW/cm^2^
Wi-Fi	0.001 μW/cm^2^

**Table 3.** The list of different microcontrollers and radio solutions considered for test setup.

**Producer**	**Type**	**f (MHz)**	**Rx (mA)**	**Tx^1^ (mA)**	**Rate (kbit/s)**	**V_CCmin_ (V)**
TI	CC2500	2,400	18	11	250	1.8
TI	Sensium	900	4.5	4.5	50	1.1
Zarlink	ZL70250	900	1.9	2	186	1.2
Nordic	nRF24L01	2,400	12	8	2,000	1.9
EM	EM9201	2,400	12	12	1,000	0.8
Sensor Dynam.	SD341	300–920	23	15	125	1.85

**Table 4.** Operation events and their power consumption.

**Segment (Figure 13)**	**Event**	**Source**	**Current (mA)**	**Time (μs)**	**Energy (nAs)**	**Portion of power consumed (%)**	
						**This work**	**Conventionnal sensor**
A	RF Start-up	RF^1^	2.3	320	736	1.9%	1.0%
B	Waiting for RF ready	RF^1^	1.75	150	262.5	0.7%	0.7%
C	Idle	RF^1^	1.55	382	232.5	0.6%	0.3%
D	PLL Calibration	RF^1^	8.5	909	7726.5	19.7%	10.6%
E	Rx mode	RF^1^	19.8	185	3663	9.4%	5.6%
F	Tx mode	RF^1^	22.5	820	18450	47.1%	30.0%
A–G	Active processing	MCU^1^	2.75	2845	7823.75	20.0%	18.2%
A–G	Low power mode	MCU^1^	1.2	65	78	0.2%	0.2%
A–G	ADC active	MCU^1^	0.88	145	127.6	0.3%	0.4%
A–G	Sensor active	Sensing element	0.045	820	36.9	**0.1%**	
A–G^2^	Sensor active	Conventional sensor	1	35000	35000		**48.6%**

Note: 1–RF = Radio Frequency communication part; MCU = Micro Controller Unit.

## References

[1] Pavlin M., Belavic D., Santo Zarnik M., Hrovat M., Možek M. (2002). Packaging technologies for pressure-sensors. Microelectron. Int.

[2] Peterson K.A., Patel K.D., Ho C.K., Rohde S.B., Nordquist C.D., Walker C.A., Wroblewski B.D., Okandan M. (2005). Novel microsystem applications with new techniques in low-temperature co-fired ceramics. Int. J. Appl. Ceram. Technol.

[3] Maluf N., Williams K. (2004). An Introduction to Microelectromechanical System Engineering.

[4] Thelemann T., Thust H., Hintz M. (2002). Using LTCC for microsystems. Microelectron. Int.

[5] Golonka L.J., Roguszczak H., Zawada T., Radojewski J., Roguszczak H., Stefanow M. (2006). LTCC microfluidic system. Int. J. Appl. Ceram. Technol.

[6] Gongora-Rubio M.R., Espinoza-Vallejos P., Sola-Laguna L., Santiago-Aviles J.J. (2001). Overview of low temperature cofired ceramics tape technology for meso-system technology (MsST). Sens. Actuat. A.

[7] Golonka L.J., Zawada T., Radojewski J., Grabowska I., Chudy M. (2005). LTCC based microfluidic system with optical detection. Sens. Actuat. B.

[8] Belavic D., Santo Zarnik M., Hrovat M., Macek S., Pavlin M., Jerlah M., Holc J., Drnovsek S., Cilensek J., Kosec M. Benchmarking Different Types of Thick-Film Pressure Sensors.

[9] Santo Zarnik M., Belavic D., Macek S. (2010). The warm-up and offset stability of a low-pressure piezoresistive ceramic pressure sensor. Sens. Actuat. A.

[10] Ong K.G., Grimes C.A., Robbins C.L., Singh R.S. (2001). Design and application of a wireless, passive, resonant-circuit environmental monitoring sensor. Sens. Actuat. A Phys.

[11] Radosavljevic G., Živanov L., Smetana W., Maric A., Unger M., Nad L. (2009). A Wireless Embedded Resonant Pressure Sensor Fabricated in the Standard LTCC Technology. IEEE Sens. J.

[12] Kouzoudis D., Grimes C.A. (2000). The frequency response of magnetoelastic sensors to stress and atmospheric pressure. Smart. Mater. Struct.

[13] Al Agha K., Bertin M.H., Dang T., Guitton A., Minet P., Val T., Viollet J.B. (2009). Which wireless technology for industrial wireless sensor networks? The development of OCARI technology. IEEE Trans. Ind. Electron.

[14] Gungor V.C., Lu B., Hancke G.P. (2010). Opportunities and challenges of wireless sensor networks in smart grid. IEEE Trans. Ind. Electron.

[15] Quevedo D.E., Ahlen A., Østergaard J. (2010). Energy efficient state estimation with wireless sensors through the use of predictive power control and coding. IEEE Trans. Signal Process.

[16] Fournier Y., Maeder T., Boutinard-Rouelle G., Barras A., Craquelin N., Ryser P. (2010). Integrated LTCC pressure/flow/temperature multisensor for compressed air diagnostics. Sensors.

[17] Birol H., Maeder T., Jacq C., Straessler S., Ryser P. (2005). Fabrication of low-temperature co-fired ceramics micro-fluidic devices using sacrificial carbon layers. Int. J. Appl. Ceram. Technol.

[18] Puers R. (1993). Capacitive sensors: When and how to use them. Sens. Actuat. A.

[19] Jiang X., Polastre J., Culler D. Perpetual Environmentally Powered Sensor Networks.

[20] Raju M. (2008). Energy Harvesting. ULP Meets Energy Harvesting: A Game-Changing Combination for Design Engineers.

